# Chronic non-freezing cold injury results in neuropathic pain due to a sensory neuropathy

**DOI:** 10.1093/brain/awx215

**Published:** 2017-08-31

**Authors:** Tom A Vale, Mkael Symmonds, Michael Polydefkis, Kelly Byrnes, Andrew S C Rice, Andreas C Themistocleous, David L H Bennett

**Affiliations:** 1 Nuffield Department of Clinical Neurosciences, University of Oxford, Oxford, UK; 2 Cutaneous Nerve Laboratory, Neurology, Johns Hopkins University School of Medicine, Baltimore, USA; 3 Pain Research Group, Department of Surgery and Cancer, Faculty of Medicine, Imperial College London, Chelsea and Westminster Hospital Campus, London, UK; 4 Pain Medicine, Chelsea and Westminster Hospital NHS Foundation Trust, London, UK

**Keywords:** sensory neuropathy, neuropathic pain, small fibre neuropathy, nerve conduction studies, peripheral nerve injury

## Abstract

Non-freezing cold injury develops after sustained exposure to cold temperatures, resulting in tissue cooling but not freezing. This can result in persistent sensory disturbance of the hands and feet including numbness, paraesthesia and chronic pain. Both vascular and neurological aetiologies of this pain have been suggested but remain unproven. We prospectively approached patients referred for clinical assessment of chronic pain following non-freezing cold injury between 12 February 2014 and 30 November 2016. Of 47 patients approached, 42 consented to undergo detailed neurological evaluations including: questionnaires to detail pain location and characteristics, structured neurological examination, quantitative sensory testing, nerve conduction studies and skin biopsy for intraepidermal nerve fibre assessment. Of the 42 study participants, all had experienced non-freezing cold injury while serving in the UK armed services and the majority were of African descent (76.2%) and male (95.2%). Many participants reported multiple exposures to cold. The median time between initial injury and referral was 3.72 years. Pain was principally localized to the hands and the feet, neuropathic in nature and in all study participants associated with cold hypersensitivity. Clinical examination and quantitative sensory testing were consistent with a sensory neuropathy. In all cases, large fibre nerve conduction studies were normal. The intraepidermal nerve fibre density was markedly reduced with 90.5% of participants having a count at or below the 0.05 centile of published normative controls. Using the Neuropathic Pain Special Interest Group of the International Association for the Study of Pain grading for neuropathic pain, 100% had probable and 95.2% definite neuropathic pain. Chronic non-freezing cold injury is a disabling neuropathic pain disorder due to a sensory neuropathy. Why some individuals develop an acute painful sensory neuropathy on sustained cold exposure is not yet known, but individuals of African descent appear vulnerable. Screening tools, such as the DN4 questionnaire, and treatment algorithms for neuropathic pain should now be used in the management of these patients.

## Teaser

Non-freezing cold injury, first described during World War I and known colloquially as ‘trench foot’, remains a common cause of disabling, career-ending pain in soldiers. Vale *et al.* show that this pain is due to an acquired sensory neuropathy, providing an evidence-based rationale for its diagnosis and treatment.

## Introduction

Just over 100 years ago ‘trench foot’ was described as a unique clinical syndrome observed in soldiers during World War I (Smith *et al.*, 1915). Trench foot was caused by exposure to cold and wet conditions, and was associated with swelling, pain and sensory disturbance of the feet. In World War II, sailors developed ‘immersion foot’ due to submersion of the feet in cold water. Non-freezing cold injury (NFCI) is the umbrella term for these conditions and is part of the spectrum of cold environmental injuries (Imray *et al.*, 2011). However, the current clinical syndrome termed NFCI is not generally associated with the same degree of tissue loss as ‘trench foot’ and ‘immersion foot’. NFCI results from exposure to temperatures between 0 and 15°C and is distinct from frostbite because tissue freezing does not occur (Ungley *et al.*, 1945; Imray *et al.*, 2011). When cold is combined with moisture the risk of injury is increased. NFCI affects individuals of African ethnicity more frequently than Caucasians (Burgess and Macfarlane, 2009). A number of phases of NFCI have been recognized (Ungley *et al.*, 1945). Acutely during the period of cold exposure, patients report sensory dysfunction in the extremities: numbness, paraesthesia and pain, as well as vasomotor disturbance (pallor) that may be followed by swelling, erythema and exacerbation of pain on rewarming of the affected limbs. Some patients develop chronic NFCI with persistent sensory symptoms, in particular chronic pain and cold hypersensitivity of the hands and feet. Despite the fact that NFCI is a recognized syndrome in military personnel (also reported in mountaineers) (Carter *et al.*, 1988), fishermen and the homeless (Wrenn, 1991; Irwin *et al.*, 1997), it remains a diagnostic challenge. There is a lack of accepted diagnostic criteria and the aetiology of chronic pain associated with NFCI is unknown. Both vascular and neuropathic causes of chronic NFCI symptoms have been posited. Animal models have suggested that cold exposure can cause injury to myelinated fibres within peripheral nerves (Denny-Brown *et al.*, 1945). A peripheral neuropathy due to NFCI has not been established in NFCI patients, probably because traditional nerve conduction studies are insufficiently sensitive due to the very distal nature of the injury (Carter *et al.*, 1988). A single case report of a patient with severe NFCI resulting in gangrene and ulceration requiring surgical debridement suggested a loss of intraepidermal nerve fibres on skin biopsy although these were not quantified (Irwin *et al.*, 1997) and the severity of this one case made it unclear if this was a core feature of NFCI.

To clearly define the neurological components of chronic NFCI we have undertaken a cross-sectional study and report the first ever cohort of NFCI patients to undergo detailed neurological evaluation. Our aims were to determine whether chronic NFCI was associated with a peripheral neuropathy, undertake detailed sensory phenotyping including grading of neuropathic pain and to establish if the pain associated with NFCI satisfies formal diagnostic criteria for neuropathic pain.

## Materials and methods

### Study participants

Study participants were enrolled into one of two studies. Actively serving British army personnel were enrolled in the ‘Neurological consequences of non-freezing cold injury’ study approved by the Ministry of Defence Research Ethics Committee (MoDREC Protocol No: 616/MoDREC/14). Ex-servicemen (veterans) were enrolled in the ‘Pain in Neuropathy Study (PiNS)’ (Themistocleous *et al.*, 2016), approved by the National Research Ethics Service of the United Kingdom (No: 10/H0706/35). Study participants were recruited from a specialist neuropathy clinic at The John Radcliffe Hospital, Oxford UK, or through self-referral via advertising. All study participants signed written consent before participation.

To be eligible, participants had to be over the age of 18 and have a history consistent with chronic NFCI. This was defined as: sensory symptoms (numbness, paraesthesias, pain and altered temperature sensibility) that began at the time of exposure to a near-freezing cold environment and persisted for at least 3 months, with no history of soft tissue freezing that would indicate a diagnosis of frostbite or frostnip. Exclusion criteria included history of a medical condition that may cause a neuropathy, pregnancy, poor or no English language skills, severe pain at recruitment from a cause other than NFCI (to prevent potential confounding influence on pain reporting as well as psychological and quality of life reported outcomes), patients with documented CNS lesions, coincident major psychiatric disorders, or patients with insufficient mental capacity to obtain informed consent or to complete questionnaires.

### Assessment of study participants

The study design consisted of a clinical assessment and investigations, quantitative sensory testing (QST), nerve conduction studies and skin biopsy, which were completed in one or two visits at the John Radcliffe Hospital, Oxford, UK. The clinical assessment consisted of a detailed history and structured neurological examination. A narrative of the study participant’s injury, including the environment where the injury was sustained, the nature of symptoms at onset and evolution of symptoms since injury, and impact the injury had on employability were recorded in a standardized manner. The participants also completed validated and standardized questionnaires that recorded the nature of their pain and the impact on their quality of life. All participants underwent screening for other potential causes of neuropathy.

### Questionnaires

The Douleur Neuropathique en 4 Questions (DN4) (Bouhassira *et al.*, 2005) and the Brief Pain Inventory (BPI) (Tan *et al.*, 2004) were completed to characterize study participant symptom profile and any limitations caused by these symptoms.

The DN4 is a screening tool for neuropathic pain, which consists of 10 components. Seven items relate to pain descriptors (burning, painful cold, electric shocks) and associated abnormal sensations (tingling, pins and needles, numbness, itching). The other three items relate to a brief bedside neurological examination in the painful area (touch hypoaesthesia, pinprick hypoaesthesia, tactile dynamic allodynia). For scoring, one is given to each positive and zero to each negative item and the total score ranges from 0 to 10. A score of ≥4 indicates that pain is likely to be neuropathic (Bouhassira *et al.*, 2005).

The BPI (Tan *et al.*, 2004) is a two-part questionnaire tool validated for assessing the severity of, and interference, caused by pain. BPI pain severity is calculated as the average of four questions that measure pain at its worst in the last week, least in the last week, average over the last week and pain at the time of questionnaire completion. Study participants score pain on an 11-point scale ranging from 0 (no pain) to 10 (pain as bad as you can imagine). Pain-related interference in activities of daily living was assessed using the 7-item Pain Interference Scale of the BPI. The scale assesses pain interference within seven domains: general activity, walking, work, relationships, mood, life enjoyment, and sleep. Study participants score these on an 11-point scale ranging from 0 (does not interfere) to 10 (completely interferes). The composite score was calculated as the mean of the seven interference items. BPI pain relief quantifies relief obtained from analgesics as percentage. Study participants also completed a body map highlighting the distribution of any pain experienced.

### Neurological examination

Structured motor and sensory neurological examination was performed in all patients to detect signs of a peripheral neuropathy (Kleyweg *et al.*, 1991; Medical Research Council Nerve Injuries Research Committee, 2010). The examination included assessment of muscle bulk by visual inspection, motor power, deep-tendon reflexes, temperature sensibility, light touch and pinprick sensation, proprioception, and vibration perception (Phillips *et al.*, 2014). These assessments were made independently from the performance of QST.

A sensory sum score was calculated for each patient, whereby a score was given for each of the four limbs and for each of four modalities: pinprick sensation, light touch sensation, vibration sensation and joint position sense/proprioception. Each of the first three modalities was first tested over the sternum and participants were asked to use this as a reference when sensation in the limbs was tested. Vibration was assessed as either present or absent rather than attempting to score the degree of deficiency, which is assessed separately in the QST protocol. Temperature sensibility was not included in the sensory sum score. Scores were assigned as follows: 0 = no abnormality; 1 = abnormality at the toe/finger; 2 = abnormality at the ankle/wrist; 3 = abnormality at the knee/elbow; 4 = abnormality at the hip/shoulder. The maximum possible score is 64 (a score of 4 for each of the four modalities in each of the four limbs).

Therefore, the sensory sum score quantifies the severity of sensory abnormalities with a higher sum score indicative of greater sensory loss. Dorsalis pedis and posterior tibial pulses were assessed.

### Evaluation of intraepidermal nerve fibre density

Intraepidermal nerve fibre density (IENFD) assessment from skin biopsy specimens is a validated and sensitive test for the detection of small fibre pathology. Punch biopsies of the skin were taken in accordance with the consensus document produced by the European Federation of Neurological Societies/Peripheral Nerve Society Guideline on the utilization of skin biopsy samples in the diagnosis of peripheral neuropathies (Lauria *et al.*, 2010*b*). Skin biopsies were not taken from participants on warfarin or those who had other contraindications to skin biopsy. Biopsies were taken 10 cm proximal to the lateral malleolus with a disposable 3-mm punch biopsy circular blade (Stiefel Laboratories Inc.) after subcutaneous lidocaine (1%, 1–2 ml) administration. The biopsy was fixed in fresh periodate-lysine-paraformaldehyde (2%) for 12 to 24 h. Tissue was then washed in 0.1 M phosphate buffer and stored for 2 to 3 days in 15% sucrose in 0.1 M phosphate buffer. After embedding in O.C.T. (Fisher Scientific), the tissue was snap-frozen and stored at −80°C.

Skin sections with 50-μm thickness were cut on a cryostat, and immunohistochemistry for protein gene product 9.5 was performed on free-floating sections using the immunoperoxidase method. Staining was performed on 24-well plates allowing reagents complete penetration of floating samples. For the bright-field method, samples were washed with Tris-buffered saline (TBS) and placed on a 5% hydrogen peroxide solution on ethanol, followed by blockade of non-specific protein binding with 4% normal donkey serum, 0.5% milk, and 0.1% Triton™ X in TBS. Primary antibody rabbit anti-protein gene product 9.5 (1:15 000; Ultraclone Ltd, or 1:800, Zytomed, Systems) was incubated overnight at room temperature. After rinsing the samples with TBS, secondary biotinylated goat anti-rabbit IgG antibody (1:400; Vector Laboratories) was used followed by addition of VECTASTAIN® ABC Kit (Vector Laboratories) for 1 h. Samples were washed and transferred to the DAB Peroxidase Substrate Kit, 3,3’-diaminobenzidine (Vector Laboratories) until a visible stain emerged. Samples were rinsed with distilled water and progressively dehydrated with ethanol. Samples were mounted using DPX mounting media. Protein gene product 9.5-immunoreactive nerve fibres crossing the basal membrane of the epidermis were counted under a 40× objective, and a measurement of the length of the sample was also obtained. The IENFD was assessed using a double bright-field microscope using established counting rules and expressed as fibres per millimetre of epidermal length, and considered abnormal if the value falls below the fifth centile for age- and gender-matched healthy controls (Lauria *et al.*, 2010*a*). Participants’ biopsies were performed, processed and analysed at The John Radcliffe Hospital, Oxford. Participants’ IENFDs were determined to be normal or abnormal relative to the published normative age and gender matched data (Lauria *et al.*, 2010*a*). Values falling below the 0.05 quantile are abnormal.

There are no published normative skin biopsy data for individuals of African descent. Age, gender, and ethnically matched skin biopsies from healthy African Americans were obtained and analysed by The Cutaneous Nerve Laboratory of The John Hopkins School of Medicine, Baltimore, USA. Healthy study participants were recruited through a John Hopkins School of Medicine approved protocol (NA_00036876) and screened for causes of neuropathy. All healthy participants were examined and determined to have a normal peripheral nerve exam and there was no history of diabetes, thyroid disease, cancer, HIV, hepatitis, vitamin B12 deficiency or significant alcohol intake (>2 units/day). Immunohistochemistry was performed, in line with the staining protocol used at the John Radcliffe Hospital, using mouse anti-human monoclonal anti-protein gene product 9.5 (1:2500; Bio-Rad). IENFD assessment was performed by K.B. at The Johns Hopkins School of Medicine, Baltimore, and by A.C.T. at The John Radcliffe Hospital, Oxford. Reliability analysis performed by Johns Hopkins, shows that Zytomed antibody, used by John Radcliffe Hospital, and Bio-Rad antibody, used by John Hopkins, are equivalent, with R^2^ = 0.92. We also performed a reliability analysis to assess IENFD assessment between A.C.T., Oxford, and K.B., Baltimore in assessing counts in 11 different individuals with each investigator blind to the other’s scores. Intra-class correlation coefficient, expressed as mean [95% confidence interval (CI)], was 0.97 (0.88–0.99) with R^2^ = 0.94. Therefore, the agreement between the laboratories was very good.

### Nerve conduction studies

Nerve conduction tests were performed in line with the American Academy of Neurology and American Association of Electrodiagnostic Medicine recommendation for the diagnosis of a peripheral neuropathy (England *et al.*, 2005). The nerve conduction tests were performed with an ADVANCE system (Neurometrix) or a Medelec Synergy EMG system (version 15.0; Viasys Healthcare UK Ltd) using conventional reusable surface recording electrodes.

Sural sensory and peroneal motor nerve conduction studies were performed in one lower extremity. If both studies were normal, no further tests were performed (Buschbacher and Orahlow, 2006). If either test was abnormal, additional nerve conductions were performed that included: ipsilateral tibial motor nerve, contralateral sural sensory nerve, peroneal motor or tibial motor nerves. The minimum case definition criterion for electrodiagnostic confirmation of neuropathy was an abnormality of any attribute of nerve conduction in two separate nerves, one of which was the sural nerve. Variables such as skin temperature, age, height, sex, and weight were measured and accounted for when interpreting nerve conduction tests.

Sympathetic skin responses were measured with patients lying supine with eyes closed in a quiet room with skin temperature at 32°C or above. Disposable Ag/AgCl electrodes were attached to the mid-palm of one hand and the mid-sole of one foot and referenced to the corresponding dorsal surface. A single 0.2 ms duration 200–300 V square-wave electrical pulse was applied to the contralateral median/tibial nerve (separately for upper and lower limbs, respectively) after an unpredictable interval to produce a sympathetic skin response. Two trials were recorded per limb to check waveform reproducibility (10 s sweep, amplifier 100 µV/division, low/high pass filter settings 0.5/100 Hz). The onset latency and peak-to-peak amplitude of the response waveform with the largest amplitude was measured.

### Quantitative sensory testing

QST is a tool that assesses the somatosensory phenotype of individuals in a standardized manner and was performed according to the protocol of the German research network of neuropathic pain (DFNS) (Rolke *et al.*, 2006*a*). QST was used to assess study participants’ ability to detect changes in thermal and mechanical stimuli and to determine the threshold at which the stimuli were felt as being painful. The investigators (T.A.V and A.C.T) underwent formal training in conducting the DFNS QST protocol at Mannheim University. Thermal detection and pain thresholds and thermal sensory limen (including paradoxical heat sensations) were measured using a Thermotest (Somedic). Mechanical detection and pain thresholds, mechanical pain sensitivity, allodynia, pressure pain thresholds, wind-up ratio, and vibration detection thresholds were measured as per the DFNS protocol. Participants were familiarized with the testing procedure on the dorsum of the forearm before all parameters were measured over the dorsum of the hand and the dorsum of the foot (S1 dermatome). Pressure pain thresholds were recorded over the thenar eminence and the arch of the foot. Vibration detection thresholds were tested over the ulnar styloid process and the medial malleolus. The QST protocol was performed unilaterally (ipsilateral hand and foot). QST data were entered into the data analysis system, Equista, provided by the DFNS. Equista transformed the raw QST data into z-scores thus normalizing for age, sex, and the body location of testing (Rolke *et al.*, 2006*b*; Magerl *et al.*, 2010). A z-score of zero is equal to the mean of the population. A score of greater or less than two standard deviations from the mean indicates gain of function or loss of function, respectively.

#### Grading of pain aetiology

Neuropathic pain is defined as ‘pain arising as a direct consequence of a lesion or disease affecting the somatosensory system’. To determine whether the chronic pain caused by NFCI is neuropathic pain, we adopted the Neuropathic Pain Special Interest Group (NeuPSIG) of the International Association for the Study of Pain (IASP) grading for neuropathic pain (Finnerup *et al.*, 2016).

Each study participant’s pain was assessed using these published criteria as below. Possible neuropathic pain must fulfil criteria 1 and 2; probable neuropathic pain must fulfil criteria 1, 2 and 3; and definite neuropathic pain must fulfil all four criteria.

1. A history suggestive of a relevant lesion or disease affecting the peripheral or central somatosensory system—a history of neuropathy symptoms including decreased sensation, positive sensory symptoms e.g. burning, aching pain mainly in the distal areas of the upper and/or lower extremities temporally related to an episode of cold exposure.
2. Pain with a neuroanatomically plausible distribution i.e. pain symmetrically distributed in the extremities—completion of body map and clinical history.
3. Demonstration of sensory signs in the same neuroanatomically plausible distribution—sensory loss in the limb extremities.
4. Demonstration of the relevant lesion or disease by at least one confirmatory test—abnormality on nerve conduction tests, IENFD or QST.

### Statistical analysis

SPSS Statistics Version 21 (IBM) and GraphPad Prism were used for statistical analysis. QST z-score data were expressed as mean (95% CI). All other data were tested for normality with the D’Agostino-Pearson normality test and by visual inspection of their distribution. Data normally distributed are reported as mean (95% CI), and data not normally distributed are reported as median with interquartile range (IQR). Spearman correlation analyses were performed to explore associations between pain severity, sensory sum score (as a measure of clinical severity), time from injury to assessment, IENFD and QST findings. Significance was set at *P* < 0.05.

## Results

### Study participant demographics

**Figure 1:**
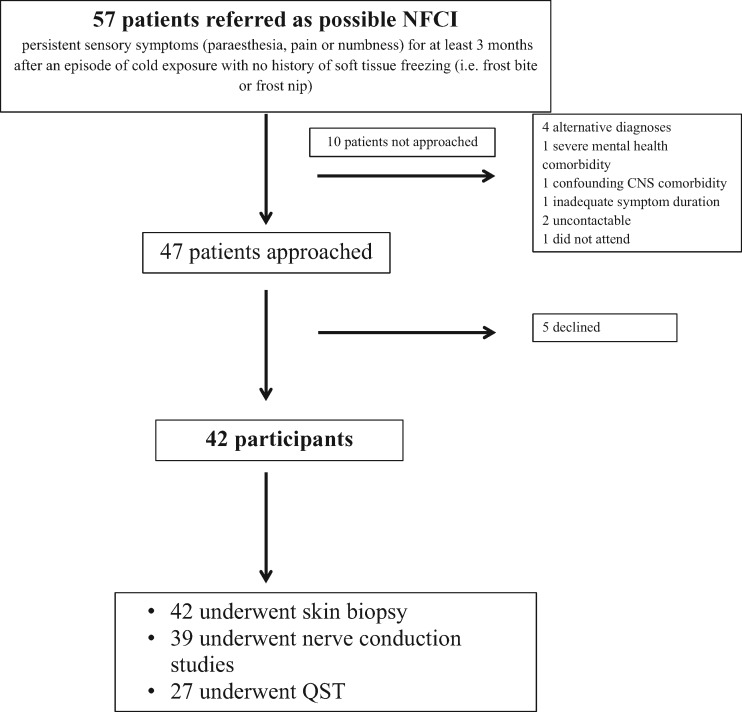
**Participant recruitment pathway.** Flow diagram of the recruitment of study participants.

Forty-two participants were recruited for assessment from a total of 57 referrals (Fig. 1). All participants were assessed by one of the study authors (T.V., A.C.T. or D.L.H.B.). The participants’ mean age was 32.4 (31.02–33.69) years and 40 (95.2%) of the participants were male. Twenty-eight participants were originally from Western Africa (66.7%), four from Southern Africa (9.5%) and six from The Caribbean (14.3%). Four participants (9.5%) were Caucasian and born in the UK. The healthy African American study participants were age- and gender-matched with a mean (95% CI) age of 29.7 years (26.1–33.3), and 88.2% of the healthy African American study participants were male.

### Narrative of injurious event

All participants suffered their injury while serving in the UK military. The onset of the participants’ symptoms was temporally related to cold exposure and the majority were associated with a concomitant wet environment (Table 1). A specific military activity, such as a cold weather exercise or tour of duty in a cold climate was cited in 97.6% of cases (Table 1). The majority of participants had been exposed to multiple cold environments without ill effect before their symptoms became persistent. Similarly, the majority of participants were re-exposed to an injurious environment after their symptoms became persistent. The main reason for re-exposure centred on the operational/training imperatives of serving in the military. Index exposures occurred throughout the world; 25 in the UK (59.5%), four in Canada (9.5%), nine in Germany (21.4%), two in Afghanistan (4.8%), one in Kosovo (2.4%) and one in the Falkland Islands (2.4%). The median time (IQR) from index exposure to time of study enrolment was 3.58 (2.73–6.19) years.

**Table 1:** Summary of exposure histories

History of exposures	*n* (%)
Cold weather	42 (100)
Wet environment	35 (83)
Previous exposure(s)	34 (81)
Duration of exposure before persistent symptom onset	
<1 h	3 (7)
<24 h	14 (33)
<1 week	16 (38)
1–2 weeks	1 (2)
2–3 weeks	1 (2)
Uncertain	7 (17)
Duration of ongoing exposure after persistent symptoms	
Immediate withdrawal	1 (2)
<1 h	1 (2)
1–24 h	6 (14)
24 h to 1 week	15 (36)
>1 week	11 (26)
Uncertain	8 (19)
Subsequent exposure(s)	36 (86)
Extremities affected	
Hands affected	40 (95)
Feet affected	42 (100)

Participants were asked to describe environmental conditions at the time they first experienced sensory symptoms. While most were unable to give precise temperatures their descriptions of the environment (e.g. presence of frost, ice or snow, in addition to the time of year and location of exposure) were consistent with temperatures associated with NFCI. Participants were asked to state how long they had been exposed before symptom onset. Most were able to say whether this was in the order of hours, days or weeks and based on their answers, the ranges provided in the table were derived. Participants were asked if they had previously and/or subsequently been exposed to such environments.

### Distribution and quality of symptoms, impact of symptoms on quality of life, and analgesic use

**Figure 2:**
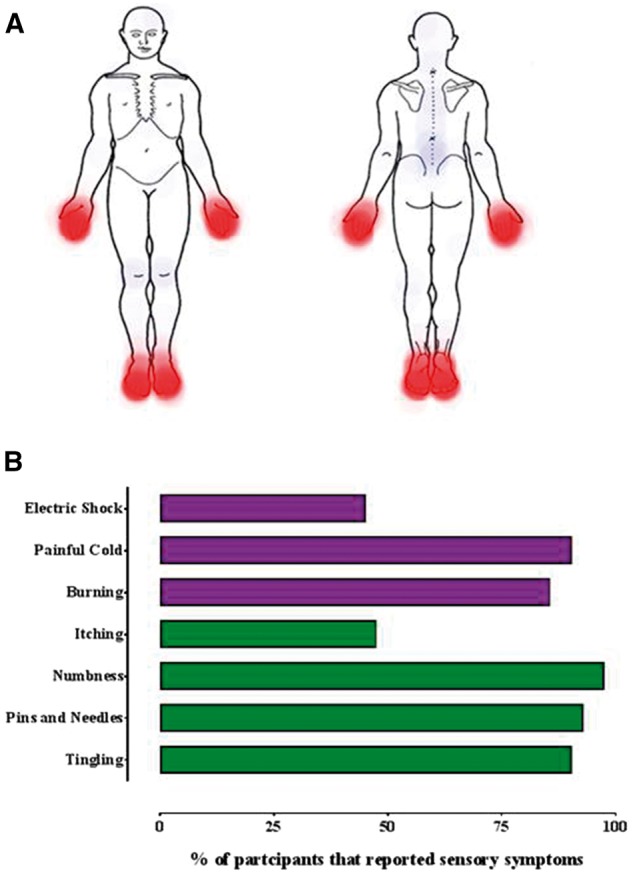
**Pain was located in the distal extremities and had neuropathic qualities.** (**A**) Composite heat map depicting the location of neuropathic pain (in red) and non-neuropathic pain (blue) reported by study participants. Study participants completed a body map highlighting the distribution of any pain experienced as part of the BPI questionnaire. (**B**) Histogram illustrating the frequency of different neuropathic pain characteristics from the DN4 questionnaire. The bars in purple (electric shock–burning) are pain descriptors. The bars in green (itching–tingling) are associated symptoms.

All participants experienced symptoms in their feet and almost all in their hands (Table 1 and Fig. 2). None of the participants suffered tissue loss, gangrene or amputation, or skin ulceration. The most prominent symptoms were chronic pain and cold hypersensitivity. All participants experienced pain limited to the hands and feet that was bilateral and symmetrical in all cases (Table 2 and Fig. 2). The median BPI pain severity was high at 6.0 (4.8–7.8). Pain qualities were neuropathic in nature, as reflected by a median DN4 score of 7.5 (6.0–8.0) and all participants scored ≥4 on the DN4 (Fig. 2). All participants experienced cold hypersensitivity (exacerbation or emergence of symptoms in cool or cold conditions that would not previously have provoked such symptoms). Subjective impairment in temperature sensibility was reported by 32 participants (76.2%), such that they had to use a more proximal part of the upper limb to test water temperature when showering, or would need to use a thermometer to test water temperature when bathing children. The chronic pain and cold hypersensitivity had a significant deleterious impact on quality of life for the study participants. The median BPI pain interference score was high at 7.2 (5.6–8.6) with impact across all aspects of daily life (Supplementary Fig. 1). For example, 73.7% of study participants reported exacerbation of symptoms when they used the fridge or freezer at home.

**Table 2:** Summary of pertinent clinical examination findings, electrophysiological recordings and IENFD

*n*	42
Bilateral impairments on sensory examination of the hands (%)	
Pinprick	37 (88.1)
Light touch	31 (73.8)
Proprioception	10 (23.8)
Vibration	5 (11.9)
Bilateral impairments on sensory examination of the feet (%)	
Pinprick	39 (92.9)
Light touch	34 (81.0)
Proprioception	20 (47.7)
Vibration	12 (28.6)
Sensory sum score	10 (6.8–12)
Absent ankle jerks (%)	4 (9.5)
IENFD (fibres/mm)	3.9 (3.5–4.4)
Sural nerve	
Amplitude (μV)	13.4 (10.4–18.6)
Conduction velocity (m/s)	48.4 (44.3–52.4)
Peroneal nerve (motor)	
Amplitude (mV) (ankle–EDB)	9.9 (7.1–13.3)
Velocity (m/s) (fibular head–ankle)	50.0 (46.2–53.8)
Sympathetic skin response (mV)	1.2 (0.7–3.15)

Nerve conduction study data were available for 39 (92.9%) study participants in the lower limbs. Sympathetic skin responses in the foot were available for 30 (71%) study participants. The nerve conduction studies and sympathetic skin response values are expressed as median (IQR). IENFD results were available for all of the study participants and expressed as mean (95% CI). EDB = extensor digitorum brevis.

NFCI is a career-limiting or career-ending injury as 35.8% of participants were medically discharged from the military at the time of assessment due to the functional limitations imposed by their injury. A further 23.8% have been discharged since assessment, or have a confirmed discharge date. Of those participants still serving in the military all were downgraded, whereby restrictions were implemented such that exposure to cold environments could be limited. Necessarily this limits deployability, potentially leading to administrative discharge. This also severely limits prospects of promotion. For all medically discharged participants, options for employment were restricted by the need to avoid further cold exposure. Of the veterans, 53.3% remained unemployed, all citing the need to work in warm environments as a factor impairing employability.

Reassuringly, 69.8% of the participants were prescribed analgesics for their pain with a median analgesic relief of 40.0% (BPI pain relief). The majority of analgesics prescribed were agents recommended for neuropathic pain. At least one neuropathic pain agent was prescribed in 62.8% of cases, with almost half of the participants prescribed amitriptyline (Supplementary Table 1).

### Structured neurological examination, nerve conduction studies and intraepidermal nerve fibre density

No participant had weakness in the upper or lower limbs. In the upper limbs 4.8% had impaired cool sensibility proximal to the wrist joint, otherwise all sensory abnormalities were restricted more distally (Table 2). In the lower limbs, there was sensory impairment across all modalities. Impairments proximal to the ankle joint were observed in 26.2% of the participants but none had impairments proximal to the knee. Ankle jerks were absent, bilaterally, in four of the participants.

Nerve conduction studies were all within age- and gender-matched normative ranges (Table 2). Sympathetic skin responses were also normal. Therefore, electrophysiological evidence of large sensory fibre deficits or impaired sympathetic efferent function was not present at the sites tested.

**Figure 3:**
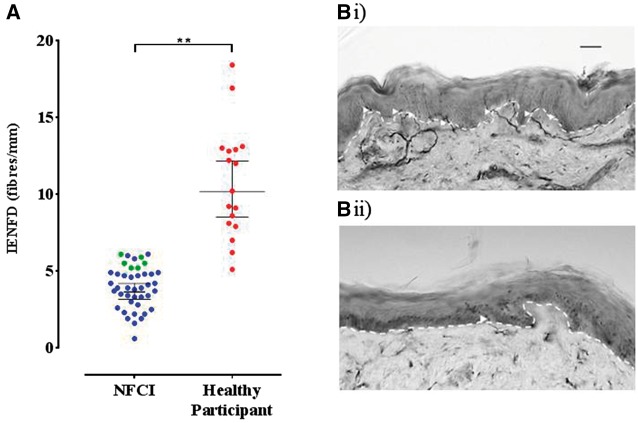
**Small fibre pathology was demonstrated on skin biopsy.** (**A**) Scatter plot and mean (95% CI) for IENFD assessment. The mean (95% CI) IENFD for NFCI participants was 3.9 fibres/mm (3.5–4.4). Blue circles represent values below the 0.05 quantile reference value for age/gender. Green circles represent values at or above the 0.05 quantile reference value. Red circles represent healthy African American study participants, whose IENFDs were all above the 0.05 quantile reference value for age and gender. (**B**) Bright field images of skin biopsies taken from the leg of (**i**) a healthy study participant; and (**ii**) participant with NFCI demonstrating PGP 9.5-immunoreactive fibres (arrow heads) crossing the basement membrane of the epidermis (dashed line). Scale bar = 40 μm. There is a clear reduction in intraepidermal nerve fibres in the participant with NFCI.

The IENFD measurements from the lower leg of our study participants showed evidence of small fibre pathology (Fig. 3 and Table 2). Compared to published age- and gender-matched normative data 85.6% of the IENFD measurements were below the 0.05 quantile. The remaining IENFD measurements, albeit above the 0.05 quantile value, were significantly lower than the median normative value. Participants’ IENFD were significantly reduced compared to healthy age- and gender-matched African American individuals (Fig. 3).

An example of a clinical assessment and patient narrative has been included in the Supplementary material.

### Quantitative sensory testing

**Figure 4:**
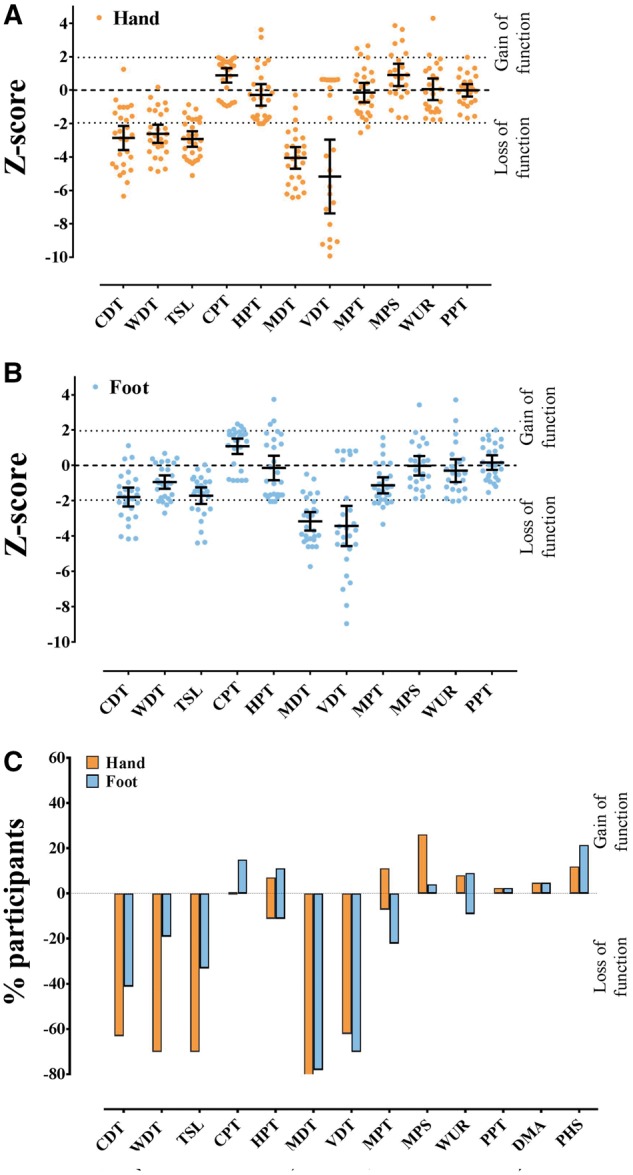
** Worse neuropathy is associated with more recent injury and more severe pain.** Sensory impairment was demonstrated on QST. Scatter plot and mean (95% CI) for QST parameters in the (**A**) hand and (**B**) foot. By comparing participant data to normative data from the DFNS a z-score can be generated, which indicates the number of standard deviations from the mean control population. Positive z-scores denote gain of function, whereas negative z-scores denote loss of function. There were significant reductions in the mean z-score in both the hand (for CDT, WDT, TSL, MDT and VDT) and foot (MDT and VDT). (**C**) Loss or gain of sensory function in the hand and foot. The *y*-axis shows the percentage of patients in each group with ‘gain’ of sensory function plotted upwards and ‘loss’ of sensory function plotted downwards i.e. z-scores that were greater or less than two standard deviations from the mean. CDT = cold detection threshold; CPT = cold pain threshold; DMA = dynamic mechanical allodynia; HPT = heat pain threshold; MDT = mechanical detection threshold; MPS = mechanical pain sensitivity; MPT = mechanical pain threshold; PHS = paradoxical heat sensation; PPT = pressure pain threshold; TSL = thermal sensory limen; VDT = vibration detection threshold; WDT = warm detection threshold; WUR = wind-up ratio.

The mean z-scores for the cold and warm detection thresholds in the hand were reduced when compared to the DFNS normative range (Fig. 4 and Supplementary Table 2). This is indicative of thermal hypoaesthesia. The mean z-scores for cold and heat pain thresholds fell within the normative range.

The mean z-scores for mechanical and vibration detection threshold in the hand were below the normative range, indicating hyposensitivity, when compared to the DFNS healthy control cohort. The z-scores for all other mechanical parameters fell within the normative range of the DFNS control data. Hyposensitivity to thermal and mechanical stimuli is indicative of loss of function in both small and large fibre modalities in the hand.

The mean z-scores for the cold detection thresholds and thermal sensory limen in the foot were at the lower limit of the DFNS normative range (Fig. 4 and Supplementary Table 2). This is indicative of thermal hypoaesthesia. The mean z-score for warm detection threshold, and cold and heat pain thresholds fell within the normative range.

The mean z-scores for mechanical and vibration detection threshold in the foot were below the DFNS normative range, indicating hyposensitivity, when compared to the DFNS healthy control cohort. The z-scores for all other mechanical parameters fell within the normative range of the DFNS healthy control data.

Thus, the QST data were almost the same in the hand and the foot, demonstrating distal loss of small and large fibre modalities.

Of the 42 study participants, 27 (64.3%) underwent QST. The remaining participants declined this investigation and were not required to provide a reason for this decision, consistent with the ethics protocol.

Only a minority of study participants exhibited gain-of-function phenomena within the pain detection parameters. Paradoxical heat sensations were elicited in 48.1% of participants (3.7% in both hand and foot, 29.6% in the foot alone and 14.8% in the hand alone) and dynamic mechanical allodynia was elicited in only one (3.7%) participant.

Mean z-scores for the foot from our participants are compared to those of patients with small fibre neuropathy and diabetic neuropathy in Supplementary Fig. 2. Data are from The Pain in Neuropathy Study (Themistocleous *et al.*, 2016).

### Grading of pain aetiology

All our participants reported chronic pain that was temporally related to an episode of cold exposure. The pain had neuropathic features exemplified by a DN4 score >4 in all participants. Pain was localized to the hands and feet and all participants exhibited sensory examination abnormalities in these areas. Therefore, all study participants satisfied the criteria for probable neuropathic pain. A diagnostic test confirmed a lesion of the somatosensory nervous system in 95.2% of participants, thus satisfying criteria for definite neuropathic pain.

### Relationships between clinical features, nerve fibre density, duration of injury and quantitative sensory testing

**Figure 5:**
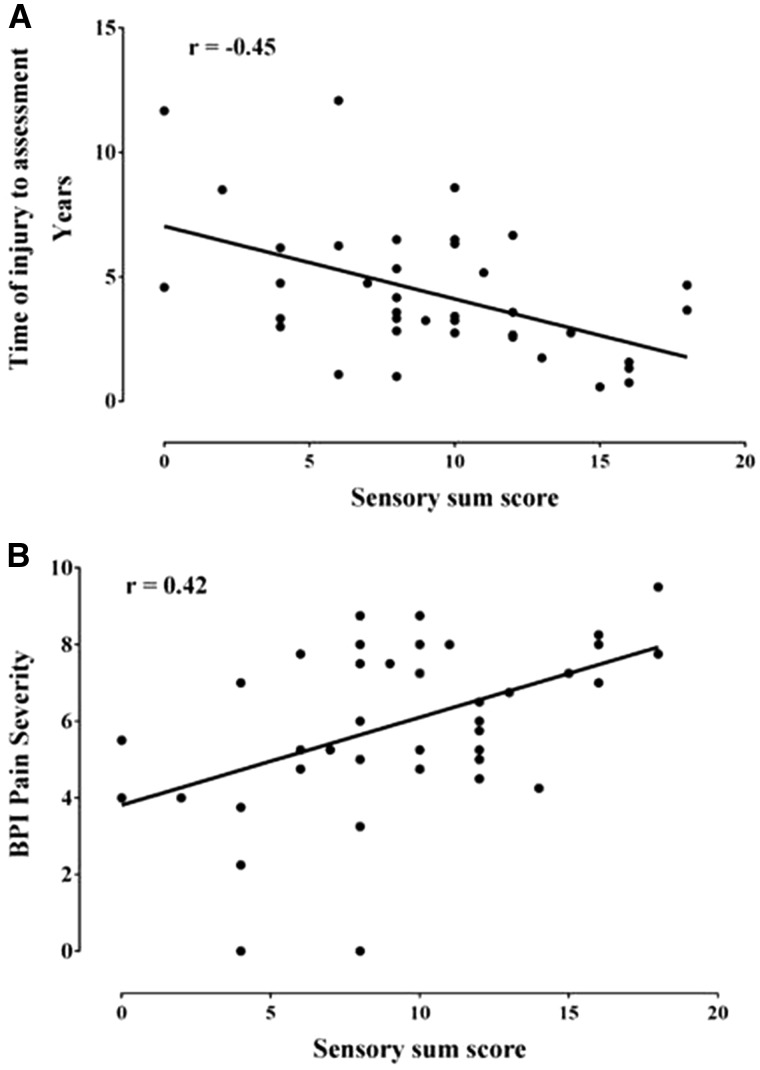
**Worse neuropathy is associated with more recent injury and more severe pain.** Scatter plot of (**A**) time of injury to assessment, and (**B**) BPI pain severity, against sensory sum score showing that sensory deficits as a measure of neuropathy severity are negatively correlated (r = −0.45, *P* = 0.003) with the duration of time that had elapsed between injury and assessment (i.e. those participants in whom a longer period of time has elapsed have less severe deficits). More severe pain was positively correlated with sensory sum score (r = 0.42, *P* = 0.005), thus neuropathic pain is associated with a more extensive neuropathy as revealed on clinical examination. Spearman correlations.

The sensory sum score correlated positively with BPI pain severity and correlated negatively with time from injury to assessment (Fig. 5 and Supplementary Table 3). Therefore, study participants with a more severe neuropathy on clinical examination reported higher pain scores, and experienced their cold injury more recently than those with a less severe neuropathy on clinical examination. The sensory sum score correlated negatively with certain QST scores for both the hand and foot (Supplementary Table 4). Therefore, study participants with a more severe neuropathy on clinical examination showed greater loss of sensation on QST parameters. IENFD did not correlate with the sensory sum score (Supplementary Table 3). There were no significant correlations between BPI pain severity and IENFD, time from injury or QST (Supplementary Tables 3 and 4). IENFD was negatively correlated with heat pain threshold recorded from both the hand and foot. This suggests that participants with lower IENFD were more sensitive to heat. Time from injury to assessment was positively correlated with mechanical detection threshold. This suggests that participants with poorer light touch sensitivity had sustained their cold injury more recently.

## Discussion

We have, for the first time, undertaken detailed neurological evaluation of patients with chronic NFCI defined as sensory symptoms (usually pain in the extremities) lasting at least 3 months after cold exposure. We report that the pain is neuropathic in origin and is due to the development of a sensory neuropathy of the extremities.

There has been conflicting evidence in the literature as to whether chronic NFCI is associated with a peripheral neuropathy. Studies have been hampered by a lack of clarity in terms of case definition and small study size (Namer *et al.*, 2008). Persistent sensory symptoms (pain, numbness, altered thermo-sensation and paraesthesia) are the predominant cause of disability in patients with chronic NFCI. A number of our findings demonstrate that chronic NFCI is a sensory neuropathy. In all of the participants there were sensory findings, most commonly impaired pin-prick sensibility in a symmetrical glove and stocking distribution and, in the more severe cases, impaired vibration sense and proprioception. Ankle jerks were absent in only a minority of patients. There was a significant positive correlation between pain severity and neuropathy severity as elicited by deficits on clinical examination. In addition, there was a significant negative correlation between the time that had elapsed between injury and assessment and neuropathy severity. It is possible that NFCI may improve with time. However, a longitudinal study is needed to verify such an observation.

Consistent with these clinical findings there were marked abnormalities on QST of the hands and feet in the form of hyposensitivity relating to thermal parameters mediated by small fibres including temperature detection, as well as hyposensitivity to stimuli activating large myelinated sensory fibres such as mechanical detection and vibration sense. A small subset of patients demonstrated hypersensitivity upon assessment of cold pain threshold or mechanical pain sensitivity. There was no significant correlation between QST findings and pain severity. However, individual QST measures were related to IENFD and the time that had elapsed between injury and assessment. Heat pain threshold was negatively correlated to IENFD. This may indicate that in those with lower IENFD the remaining nociceptors are sensitized to heat with complex outcomes in terms of the final pain perception. Mechanical detection threshold was positively correlated to the time that had elapsed between injury and assessment, thus the improvement in light touch sensitivity may be a surrogate for neuropathy recovery. It is noteworthy that despite uniformly describing cold hypersensitivity, only 15% of our cohort demonstrated gain of function in cold pain sensitivity at the foot. None of these patients exhibited loss of cold detection function in the same area, a feature seen in 41% of our cohort. The 95% confidence intervals for thermal pain thresholds are wide in the DFNS healthy control cohort (Rolke *et al.*, 2006*b*). It is plausible that in those patients who have severely impaired cold detection, the QST protocol may not be sufficiently sensitive to detect cold hypersensitivity. In addition, a brief and localized cold stimulus in the laboratory is different from that of more prolonged and extensive environmental exposure, which elicits the patients’ symptoms.

We have used the DFNS database as a normative dataset for analysis of the QST data. This has advantages in terms of robust comparisons to a large gender- and age-matched control cohort. However, one potential disadvantage is that these normative data relate to Caucasians and many of the NFCI study participants are of African descent. There have been few direct comparisons of QST from different ethnic groups. De Kruijf *et al.* (2016) reported a small reduction in cold detection sensitivity in those with black skin. In our experience, in a control Indian population (unpublished data) or mixed South American population (von Bischhoffshausen *et al.*, 2017) there was either no, or only minor (only in mechanical pain sensitivity) differences, respectively, in comparison to the DFNS control cohort. We do not feel, therefore, that such ethnic differences would be responsible for the large changes that we observed on QST.

A recent analysis of QST profiles in a large cohort of patients with peripheral neuropathic pain found that they could be broadly grouped into three clusters (Baron *et al.*, 2017). The overall profile in NFCI patients parallels the ‘sensory loss’ cluster in showing loss of small and large fibre function (present in 42% of patients suffering from peripheral neuropathic pain). The pattern of the QST profile in NFCI is distinct from small fibre neuropathy, given the involvement of large fibre modalities, and more closely resembles the pattern seen in diabetic neuropathy (Supplementary Fig. 2).

Nerve conduction studies were normal and did not show a reduction in sensory nerve action potentials. This seems a paradox given the clear sensory deficits described above. One potential explanation is that, given the superficial nature of NFCI, it is only the most distal sensory terminals that are affected, which are not interrogated by conventional neurophysiology. This would also provide an explanation as to why ankle jerks were only absent in a minority of cases. Indeed a case report of NFCI sustained while mountaineering showed that the most marked changes were in the most distal segments of nerves that were either electrically stimulated on the dorsum of the foot or via activation of Pacinian corpuscles (Carter *et al.*, 1988). When investigating potential NFCI the diagnosis of sensory neuropathy should not be discounted in the face of normal sensory nerve conduction studies. Additional investigations such as skin biopsy and QST should be performed.

Skin biopsy demonstrated a reduction in IENFD, and in 86% of participants this was below the 0.05 quantile of normative data. Furthermore, given that published normative data do not specify ethnicity and the majority of our patients were of West African or Caribbean ethnicity, we compared the IENFD in the NFCI cohort to healthy individuals of African American ethnicity and there was also a significant reduction. These findings therefore confirm structural injury to small sensory nerve fibres (predominantly nociceptors and thermoceptors) that innervate the epidermis. IENFD was not related to time of injury from assessment, nor the clinical examination. Therefore, histological assessment of small nerve fibres is helpful in confirming the diagnosis of NFCI, but does not relate to severity of neuropathy, pain nor clinical recovery in our cohort.

The pain associated with NFCI was localized to the hands and feet and all study participants reported cold hypersensitivity. Pain descriptors were typical for neuropathic pain and in all of our participants with chronic NFCI pain was either probably or definitely neuropathic in origin according to recently revised grading criteria (Finnerup *et al.*, 2016). The cold hypersensitivity, which was reported by all participants and was also apparent on QST in a subset of patients, is a striking component of NFCI. A ‘painful cold’ quality of sensory abnormality is a discriminating symptom in assessing patients with neuropathic pain (and is included in the DN4 questionnaire); however, marked provocation of symptoms by a cold environment is relatively rare. One striking example is the cold pain induced by the chemotherapy agent, oxaliplatin, the sequel of which can be very reminiscent of NFCI (for instance needing to wear gloves to remove objects from the fridge) (Ventzel *et al.*, 2016). The QST profiles for patients with NFCI and oxaliplatin-induced neuropathy are different (Binder *et al.*, 2007), and future studies will be needed to look at commonalities between these conditions.

Pain induced by NFCI was chronic and disabling. All participants had a change in employment status as a consequence of NFCI, usually due to their inability to tolerate a cold environment. In addition, the chronic pain adversely affected their quality of life, a substantial impact in otherwise young and healthy individuals.

The aetiology of NFCI has not yet been established. Our focus has been on neurological sequelae; however, abnormalities in vascular responsiveness to cold have also been reported in human NFCI (Daanen, 2003; Golden *et al.*, 2013). Differences have also been noted in cold-acclimatized individuals versus non-acclimatized (Daanen, 2003). It is striking that in some cases a single exposure to cold can lead to sensory afferent degeneration. Unlike frostbite, freezing does not occur in NFCI, so axon degeneration is not due to tissue disruption by ice crystals. The vasoconstriction and vascular injury elicited by cold could have a role, for instance through causing ischaemia of axons (Jia and Pollock, 1997). There have been attempts to develop preclinical animal models, for instance immersion of a limb in cold water, or exposure of a nerve trunk to cold. It is not clear how well such models replicate human NFCI and reports that myelinated axons are particularly vulnerable are not consistent with the striking small fibre deficits that we found (Nukada *et al.*, 1981).

There are many factors that could contribute to the vulnerability of sensory afferents in NFCI. Many such afferents terminate superficially in skin (in contrast to motor axons) and they selectively express ion channels such as TRPM8 and TRPA1, which act as cold transducers (Lolignier *et al.*, 2016). It has been shown in mice that the cold hypersensitivity resulting from oxaliplatin administration is subserved by enhanced responsiveness of TRPA1, the effect being abolished by administration of TRPA1 antagonists or TRPA1 deficiency (Zhao *et al.*, 2012). However, one study in humans investigated the response to application of TRPA1 and TRPM8 agonists in individuals that had experienced NFCI and reported that there was no evidence of sensitization (Namer *et al.*, 2008). Unlike other neuronal classes, nociceptors are activated by cold, which could potentially lead to excitotoxicity, as has been illustrated through the finding that sodium channel variants can lead to small fibre neuropathy (Rolyan *et al.*, 2016). Nerve injury may bring about changes in the direct transduction of cold stimuli, but also in how resulting responses are modulated. For example the role of potassium currents in modulating the excitability of injured cold sensing neurons has been studied, recently, in a mouse model of neuropathic pain (González *et al.*, 2017). Individuals of African ethnicity are particularly vulnerable to NFCI and the majority of participants in our cohort were African or Caribbean (Burgess and Macfarlane, 2009; Nagarajan, 2015). The basis of this vulnerability is not yet clear. There are ethnic differences in vascular responses, for instance Africans show reduced cold-induced vasodilatation that may result in increased vulnerability to NFCI (Daanen, 2003; Eglin *et al.*, 2013). There may be genetic differences in the complement of ion channels expressed in sensory neurons (Kim *et al.*, 2006). Finally, there may be ethnic differences in the regenerative capacity of sensory neurons. For instance, following topical capsaicin treatment to the skin, sensory axon regeneration has been shown to be significantly slower in African Americans (Polydefkis *et al.*, 2006).

All the patients that we reviewed experienced their injury while employed in the armed forces. Armed service personnel may be particularly vulnerable to this form of injury because they are unable to remove themselves from the environment. The type and severity of the initial injury is distinct from the reports of ‘trench foot’ and ‘immersion foot’ in soldiers during World Wars I and II. These were often severe injuries with soft tissue swelling, skin ulceration and, in a small number of cases, surgical debridement/amputation was required. None of our patients required surgical intervention and it is important to not only focus on tissue appearance or perfusion but also sensory symptoms when making an assessment of potential NFCI in the field. Although all participants in the current study suffered from pain, it is possible that there are individuals who have acquired a small fibre neuropathy after cold exposure, but who do not experience pain. Such individuals would not have been excluded, but none were referred. This may be because individuals with less distressing symptoms may not present or because such individuals are not referred. A prospective study may address this potential bias in recruitment.

Our finding that NFCI is a sensory neuropathy resulting in neuropathic pain enables the development of clear diagnostic criteria for chronic NFCI that can be applied to future prospective studies as well as being used in clinical practice. Care pathways should be aimed at early recognition to prevent prolonged and repeated exposure, screening for neuropathic pain (for instance using tools such as the DN4 questionnaire) and finally provision of therapeutics targeting neuropathic pain.

## Supplementary Material

Supplementary Table S1

Supplementary Table S2

Supplementary Table S3

Supplementary Table S4

Supplementary Table Legends

Supplementary Figure S1

Supplementary Figure S2

Supplementary Data

## Abbreviations

BPI: Brief Pain Inventory
DFNS: Deutschen Forschungsverbund Neuropathischer Schmerz
IENFD: intraepidermal nerve fibre density
NFCI: non-freezing cold injury
QST: quantitative sensory testing
