# Supplementary material for: Chronic non-freezing cold injury results in neuropathic pain due to a sensory neuropathy
Source: Brain. 2017 Aug 31;140(10):2557–69. doi: 10.1093/brain/awx215 (PMC5841153; doi:10.1093/brain/awx215)
Supplement: Supplementary Table S1 [file awx215_supp_table1.pdf]

**Analgesic use**	**30/42 (69.8%)**
- Monotherapy	17 (56.7%)
- Multiple analgesics	13 (43.3%)
Tricyclic Antidepressants	
- Amitriptyline	14 (46.7%)
- Nortriptyline	3 (10.0%)
Gabapentinoids	
- Gabapentin	7 (23.4%)
- Pregabalin	8 (26.7%)
NSAIDS	
- Naproxen	3 (10.0%)
- Meloxicam	1 (3.3%)
Opioid	
- Oral morphine	1 (3.3%)
- Codeine containing	6 (20.0%)
- Tramadol	4 (13.3%)
Topical therapies	
- Ibuprofen	1 (3.3%)
- Lidocaine	3 (10.0%)
Other	
- Carbamazepine (prescribed for headache)	1 (3.3%)
BPI Pain relief	40% (17.5-60.0%)

Supplementary Table 1
