# Supplementary material for: Chronic non-freezing cold injury results in neuropathic pain due to a sensory neuropathy
Source: Brain. 2017 Aug 31;140(10):2557–69. doi: 10.1093/brain/awx215 (PMC5841153; doi:10.1093/brain/awx215)
Supplement: Supplementary Table S2 [file awx215_supp_table2.pdf]

	Foot (Mean skin temperature 28.6˚C)				Hand (Mean skin temperature 31.2 ˚C)			
**Modality**	**Absolute Value**	**Z Score**	**% with gain of function**	**% with loss of function**	**Absolute Value**	**Z score**	**% with gain of function**	**% with loss of function**
**CDT**	22.76 °C (19.81-25.71)	-1.79 (-2.33 - -1.25)	0	41	24.82 °C (22.13 – 27.51)	-2.86 (-3.58 - -2.14)	0	63
**WDT**	40.18 °C (38.58 – 41.78)	-0.9433 (-1.32 - -0.56)	0	19	40.53 °C (38.65 – 42.41)	-2.61 (-3.16 - -2.07)	0	70
**TSL**	20.05 °C (15.74 – 24.36)	-1.71 (-2.18 - -1.24)	0	33	17.14 °C (13.00 – 21.27)	-2.93 (-3.39 - -2.46)	0	70
**CPT**	20.36 °C (16.68 – 24.05)	1.08 (0.65 – 1.52)	15	0	19.37 °C (15.46 – 23.28)	0.89 (0.46 – 1.32)	0	0
**HPT**	45.91 °C (44.23 – 47.59)	-0.08 (-0.79 – 0.63)	11	11	45.60 °C (43.80 – 47.40)	-0.28 (-0.93 – 0.36)	7	11
**MDT**	196.70 mN (105.00 – 288.40)	-3.13 (-3.67 - -2.59)	0	78	66.99 mN (34.60 – 99.37)	-4.06 (-4.70 - -3.41)	0	89
**VDT**	5.11 (4.34 – 5.89)	-3.43 (-4.56 - -2.29)	0	70	5.77 (4.92 – 6.61)	-5.29 (-9.11 – 0.61)	0	62
**MPT**	352.80 mN (259.00 – 446.70)	-1.14 (-1.60 - -0.69)	0	22	192.10 mN (117.30 – 266.90)	-0.15 (-0.73 – 0.43)	11	7
**MPS**	2.55 (-0.35 – 5.44)	-0.16 (-0.78 – 0.46)	4	0	5.64 (1.32 – 9.96)	0.90 (0.23 – 1.57)	26	0
**WUR**	3.48 (1.38 – 5.59)	-0.34 (-0.96 – 0.28)	9	9	2.73 (1.63 – 3.84)	0.06 (-0.59 – 0.70)	8	0
**PPT**	609.10 kPa (513.20 – 705.00)	0.17 (-0.25 – 0.58)	0	0	498.70 kPa (435.50 – 561.90)	-0.01 (-0.38 – 0.36)	0	0

Supplementary table 2
