# Supplementary material for: Chronic non-freezing cold injury results in neuropathic pain due to a sensory neuropathy
Source: Brain. 2017 Aug 31;140(10):2557–69. doi: 10.1093/brain/awx215 (PMC5841153; doi:10.1093/brain/awx215)
Supplement: Supplementary Table S3 [file awx215_supp_table3.pdf]

	BPI Pain Severity	Time from injury to assessment	IENFD (fibres/mm)
**Sensory sum score**	0.42**	-0.45**	0.06
**BPI Pain Severity**		-0.10	-0.12
**Time of injury to assessment**			-0.22

Supplementary table 3
