# Supplementary material for: Chronic non-freezing cold injury results in neuropathic pain due to a sensory neuropathy
Source: Brain. 2017 Aug 31;140(10):2557–69. doi: 10.1093/brain/awx215 (PMC5841153; doi:10.1093/brain/awx215)
Supplement: Supplementary Table S4 [file awx215_supp_table4.pdf]

A)

**Hand**	**CDT**	**WDT**	**TSL**	**CPT**	**HPT**	**MDT**	**VDT**	**MPT**	**MPS**	**WUR**	**PPT**
**Sensory sum score**	-0.52**	-0.64**	-0.62**	-0.24	-0.47*	-0.73**	-0.07	-0.50**	-0.24	-0.13	-0.47*
**BPI Pain Severity**	-0.12	0.01	-0.12	-0.02	0.13	-0.18	-0.22	-0.15	-0.12	-0.07	-0.15
**IENFD (fibres/mm)**	-0.14	-0.36	-0.20	-0.32	-0.57**	-0.08	0.10	-0.14	0.02	-0.07	-0.19
**Time of injury to assessment**	0.05	0.36	0.31	-0.16	0.28	0.54**	0.09	0.23	0.14	0.41*	0.05

B)

**Foot**	**CDT**	**WDT**	**TSL**	**CPT**	**HPT**	**MDT**	**VDT**	**MPT**	**MPS**	**WUR**	**PPT**
**Sensory sum score**	-0.60**	-0.40*	-0.62**	-0.30	-0.48*	-0.82**	-0.26	-0.62**	-0.44*	-0.08	-0.20
**BPI Pain Severity**	-0.20	-0.02	-0.16	-0.05	-0.13	-0.23	-0.22	-0.05	-0.15	-0.10	-0.07
**IENFD (fibres/mm)**	-0.09	-0.14	-0.16	-0.07	-0.40*	0.07	0.08	-0.16	-0.07	-0.14	-0.19
**Time of injury to assessment**	0.23	0.16	0.24	-0.18	0.33	0.57**	0.33	0.26	0.14	0.18	-0.10

Supplementary table 4
