# Supplementary material for: Chronic non-freezing cold injury results in neuropathic pain due to a sensory neuropathy
Source: Brain. 2017 Aug 31;140(10):2557–69. doi: 10.1093/brain/awx215 (PMC5841153; doi:10.1093/brain/awx215)
Supplement: Supplementary Table Legends [file awx215_supp_table_legends.pdf]

Supplementary Table 1

Summary of analgesic use. BPI pain relief expressed as median (IQ). BPI pain relief quantifies relief obtained from analgesics as a percentage, with 0% indicating no pain relief and 100% indicating complete pain relief.

Supplementary Table 2

QST results for hand and foot. Mean absolute values and mean Z scores (95% CI) are shown, as well as the percentages of participants with gain or loss of function.
mN - milliNewton; kPa - kilopascal.

Supplementary Table 3

Summary of correlations between sensory sum score, BPI pain severity, IENFD, and time from injury to assessment
Spearman correlation
Symbols reflect the significant correlations between the respective groups
* P < 0.05, ** P < 0.01
BPI - Brief Pain Inventory; IENFD – Intra-epidermal nerve fibre density

Supplementary Table 4

Summary of correlations between BPI pain severity, IENFD, clinical scores and time of injury of assessment to QST for the A) hand and B) foot
Spearman correlation
Symbols reflect significant correlations between the respective groups
* P < 0.05, ** P < 0.01

BPI - Brief Pain Inventory; IENFD – Intra-epidermal nerve fibre density; QST – Quantitative sensory testing; CDT, cold detection threshold; CPT, cold pain threshold; HPT, heat pain threshold; MDT, mechanical detection threshold; MPS, mechanical pain sensitivity; MPT, mechanical pain threshold; PPT, pressure pain threshold; TSL, thermal sensory limen; VDT, vibration detection threshold; WDT, warm detection threshold; WUR, wind-up ratio.

Supplementary Table 4

Summary of correlations between BPI pain severity, IENFD, clinical scores and time of injury of assessment to QST for the A) hand and B) foot

Spearman correlation

Symbols reflect significant correlations between the respective groups

* $P < 0.05$, ** $P < 0.01$

BPI - Brief Pain Inventory; IENFD – Intra-epidermal nerve fibre density; QST – Quantitative sensory testing; CDT, cold detection threshold; CPT, cold pain threshold; HPT, heat pain threshold; MDT, mechanical detection threshold; MPS, mechanical pain sensitivity; MPT, mechanical pain threshold; PPT, pressure pain threshold; TSL, thermal sensory limen; VDT, vibration detection threshold; WDT, warm detection threshold; WUR, wind-up ratio.

Supplementary figure 1

Scatter plot and median (IQ) for BPI pain interference score and individual scores within the 7 domains of daily life- general activity, walking, work, relationships, mood, life enjoyment, and sleep.

Supplementary figure 2

Comparison of mean z-scores for all QST parameters across three different causes of peripheral nerve injury recorded from the feet. Study participants diagnosed with small fibre neuropathy (n=25) or diabetic neuropathy (n=191) were recruited through the Pain in Neuropathy Study. Individuals diagnosed with small fibre neuropathy demonstrate a reduction in thermal detection thresholds. In contrast individuals diagnosed with diabetic neuropathy and study participants with NFCI demonstrate reduction in both thermal and mechanical thresholds.

WDT- Warm Detection Threshold, CDT – Cold Detection Threshold, TSL - Thermal Sensory Limen; Cold Pain Threshold; Heat Pain Threshold.
MDT- Mechanical Detection Threshold; VDT- Vibration Detection Threshold; MPT- Mechanical Pain Threshold; MPS- Mechanical Pain Sensitivity; WUR- Wind-up ratio; PPT- Pressure Pain Threshold.
