# Supplementary figures and images for: Chronic non-freezing cold injury results in neuropathic pain due to a sensory neuropathy
Source: Brain. 2017 Aug 31;140(10):2557–69. doi: 10.1093/brain/awx215 (PMC5841153; doi:10.1093/brain/awx215)

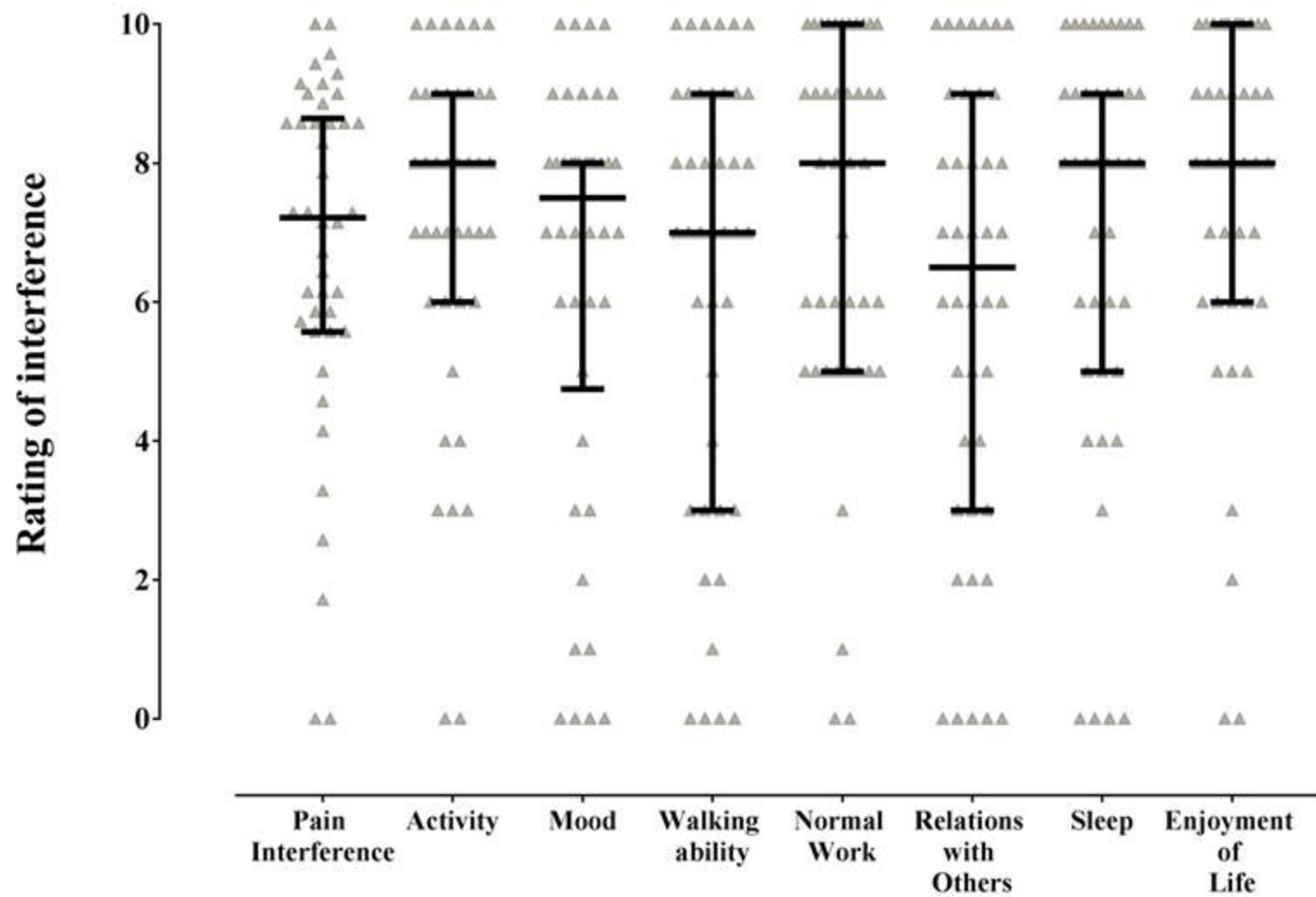

Supplementary figure 1

Supplement: Supplementary Figure S1 [file awx215_supp_figure1.pdf]

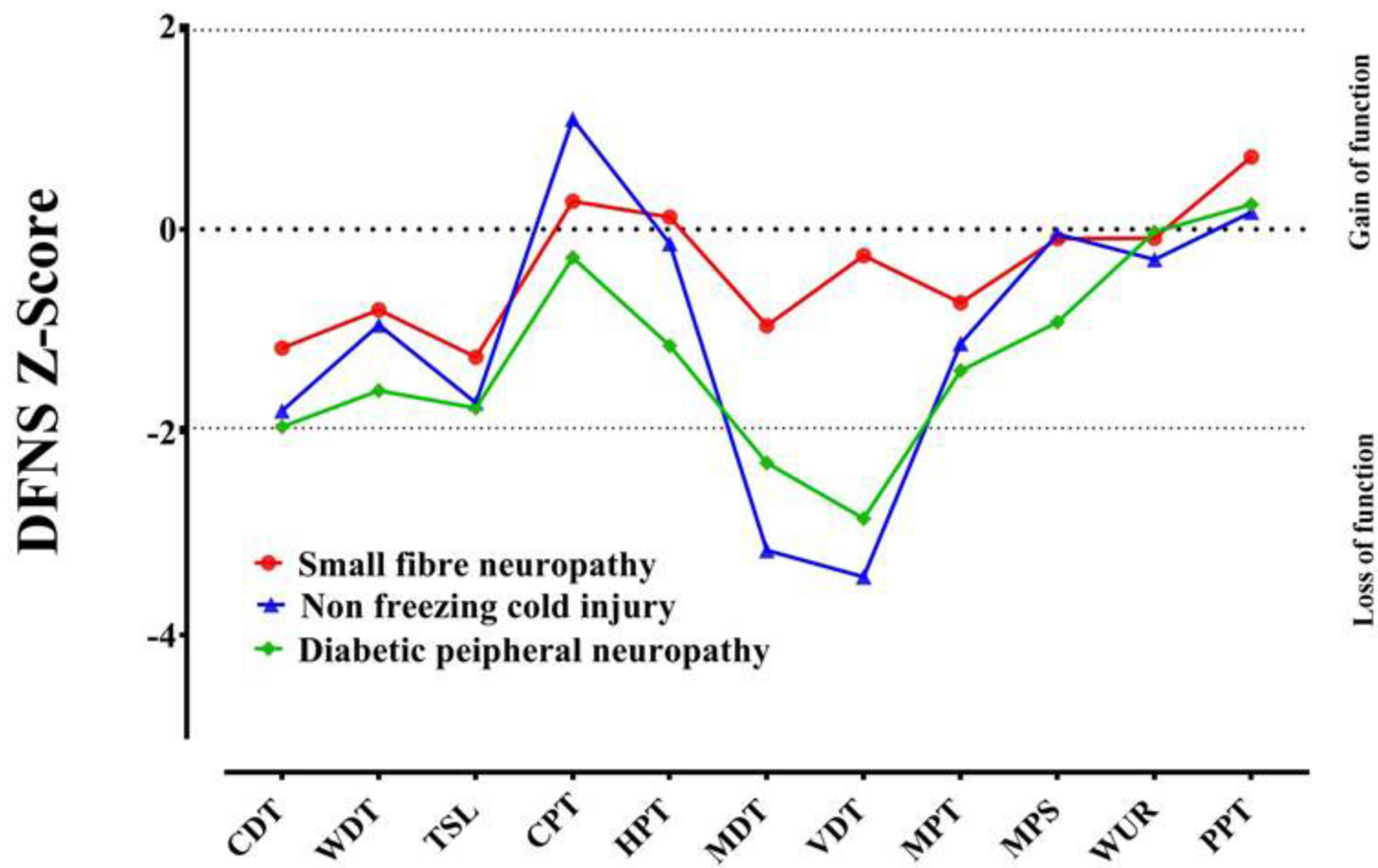

Supplementary Figure 2

Supplement: Supplementary Figure S2 [file awx215_supp_figure2.pdf]
