# Supplementary material for: Chronic non-freezing cold injury results in neuropathic pain due to a sensory neuropathy
Source: Brain. 2017 Aug 31;140(10):2557–69. doi: 10.1093/brain/awx215 (PMC5841153; doi:10.1093/brain/awx215)
Supplement: Supplementary Data [file awx215_supp_data.pdf]

## Example of an individual patient narrative

A 37 year old man, originally from Ghana, presented with sensory symptoms affecting the hands and feet, including pain, numbness and paraesthesia. He was unemployed at the time of assessment, having been discharged from the UK armed forces on medical grounds.

He dated symptom onset to a leadership course in the southern United Kingdom five years before assessment. The course lasted three weeks. The weather was cold, estimated at below 10 degrees Celcius (50 degrees Fahrenheit) and it was frequently raining. He had to undertake numerous river crossings. He was also required to crawl through a waterlogged tunnel. He did not experience any symptoms for the first week, but after this time he noted numbness of his hands and feet. Whilst numbness was the predominant symptom, he also experienced pins and needles to a lesser extent, as well as discomfort rather than pain. The numbness persisted throughout the rest of the course. On the last day he had to stand in a river for up to 40 minutes. Following completion of the course the numbness persisted for two months, whether he was in warm or cold conditions. There was some improvement after this time, but he has never appreciated normal sensation ever since this time.

Four years before assessment he deployed on a winter tour to Afghanistan for several months. Whilst performing outdoor guard duties in the cold, he noted a worsening of the numbness in his finger tips and toes. He reported these symptoms and was transferred to work inside a warm tent for the remainder of the tour. There was a little improvement in his numbness. On return to the UK he reported to his medical officer and a referral was made to the Cold Injuries Clinic at The Institute of Naval Medicine, Gosport, UK.

Whilst waiting for his appointment he continued to be put on outdoor duties and exercises, each lasting one to two weeks. Each exposure was associated with an exacerbation of symptoms, with pain clearly becoming a more prominent symptom, which he described as stabbing in nature. Over time he noted that whilst his symptoms were worsened on exposure to cool/cold environments, they were still present even in warm or hot environments, including during a visit home to Ghana.

He does not feel confident in discerning temperature with his hands, to the extent that he uses a bucket of water to wash himself as he is worried he will burn himself in a shower. He uses his elbow to tell the temperature of the bucket water. He avoids the cold aisle at the

supermarket due to cold hypersensitivity. He reported that his sleep was “distorted” and that he no longer enjoyed life.

There is no relevant past medical history and no family history of painful conditions or neurological disease. At the time of assessment he was taking pregabalin 300mg BD, amitriptyline 75mg at night and omeprazole. He reported only minimal analgesic effect (20% improvement recorded in his Brief Pain Inventory).

On examination his gait was antalgic. Cranial nerve examination was normal. There was no weakness in the arms or legs and coordination was normal. Sensory examination of the upper limbs revealed absent pinprick and light touch sensation to the level of the metacarpophalangeal joints, absent cool sensibility in the fingers, absent vibration sense at the level of the distal interphalangeal joint on the right and normal joint position sense (responses were hesitant, but correct). In the lower limbs pinprick sensation was absent to the level of the mid foot, bilaterally, and light touch to the level of the forefoot. Vibration sense was present at the right great toe and at the left metatarsophalangeal joint. Joint position sense was present for large, but not small, movement of the great toe.

Nerve conduction studies revealed normal responses from the lower limbs.

Skin biopsy showed a reduced intraepidermal nerve fibre density of 1.9 fibres/mm (median age matched reference value 10.3 fibres/mm, 0.05 quantile value 5.2 fibres/mm).
